# Postoperative mortality after a hip fracture over a 15-year period in Denmark: a national register study

**DOI:** 10.1080/17453674.2020.1744982

**Published:** 2020-04-01

*Sir,—*I have, with great interest, read the study by Gundel et al. (2020) that gives national results on hip fracture care in Denmark. Given the high-quality national registers in Denmark, my expectations were high. The fact that the 1-year-mortality was found to be high, 27%, and unchanged during a period of 15 years increased my attention further. Regrettably, my enthusiasm died down (no pun intended). The authors did not discuss any reason for the high mortality in terms of public health, quality of hospital and elderly care, psychological factors post-trauma leading to loss of self-preservation, etc. A Western country like Denmark with easily accessible public healthcare ought to have a lower mortality rate after hip fracture, in particular when fracture patients of all ages are included. The same mortality rate was found when studying only Finnish patients over 65 years (Panula et al. 2011). A 2011 urban cohort of all hip fracture patients over 20 years, in Malmö, Sweden, had a mortality rate of 24% (Hansson et al. 2015). A recent meta-analysis suggested 22% to be an expected mortality rate (Downey et al. 2019).

Instead, the authors choose to investigate whether surgical methods have any association with the risk of death. They found “operation type other than total hip arthroplasty was … associated with postoperative mortality.” Being one of the authors cited in this question, I would like to stress the pronounced selection bias when using surgical methods as predictors. In our paper (Hansson et al. 2019), we did not discuss the difference in mortality between total hip arthroplasty and hemiarthroplasty as a clinically relevant result. We clearly explained this finding as a result of confounding and nothing else. Drawing conclusions and clinical perspectives on THA being associated with a reduced risk of dying reveals a lack of orthopedic experience in the author group. THA is recommended for healthier, active patients with good chances of long-term survival. This is only touched upon in the Danish paper.

In addition, when comparing different operations, the authors have chosen “closed reposition” as reference. The options compared with closed reduction are “open reposition, external fixation, internal fixation, hemiarthroplasty, arthroplasty” and “other.” It makes no sense to use closed reposition either as a procedure of its own, or as the reference. Surgical treatment of a hip fracture starts with either closed or open reduction and is then followed by internal fixation. Arthroplasty is the other main alternative. External fixation is extremely uncommon, here used in less than 1 per 1,000 of the patients, and may represent either multi-trauma cases or miscoding. Table 2 rewritten with internal fixation as reference would have provided the reader with relevant comparisons.

The authors state: “Further studies are needed to shed light on whether the type of operative procedure influences the postoperative mortality following HF surgery and whether the prognosis of specific comorbid elderly patients can be modulated by choosing procedures with less trauma and thus less surgical stress.” This has been a major aim for numerous studies during the last 25 years (Rogmark and Johnell 2006), comparing the more strenuous arthroplasty procedure with a quick pinning or screwing of a displaced femoral neck fracture. Arthroplasty led to less pain, better function and health-related quality-of-life, and fewer reoperations, without any clear risk of increased mortality. Sometimes, more is more.

Besides pointing out that those who are men, sick, and old have a worse prognosis after a hip fracture – well-known factors (Dahl 1980) – the paper is a good example of how an epidemiology paper has to be written in cooperation with researchers who are in clinical practice and with knowledge of the disease-specific literature.

I hope the Köge group will continue to study outcomes after hip fracture, to the benefit of elderly Danes, and to improve Danish healthcare further. When doing so, I hope they involve co-workers with specific clinical expertise.

**Cecilia Rogmark**

Skane University Hospital/Lund University Malmö, Sweden

*E-mail:* cecilia.rogmark@skane.se

*Sir,—*Thank you for the opportunity to comment on the letter from Associate Professor Cecilia Rogmark, regarding our paper “Postoperative mortality after a hip fracture over 15 years in Denmark: a national register study.”

We appreciate that Dr Rogmark took her time to read and comment on our paper and the results, and the scientific debate about how to improve postoperative mortality. Below are our comments to the remarks by Dr Rogmark.

Our primary aim with this study was to investigate whether there had been any change in the mortality of Danish hip fracture patients over a period of 15 years. These data have not previously been reported for Danish hip fracture patients. With the focus on enhanced recovery after surgery in other surgical patient groups, we see our data as relevant in the debate about lowering postoperative mortality and complications.

With these epidemiological data, we are not able to draw any certain conclusion as to why mortality has not changed over time. We acknowledge that the quality of hospital and elderly care could likely influence mortality over a 15-year period, which could have been interesting to include in the analysis. Other clinical factors with potential importance to the outcome after hip fracture surgery are early epidural/type of anesthesia (Van Waesberghe et al. 2017) and time to surgery. Unfortunately, these data were not available in the study. Moreover, we agree that we could have done an extended analysis on the potential change in number of comorbidities in the population over these 15 years.

As life expectancy increases and patients are living with more comorbidities (World Health Organization 2015), this could partly explain why we did not see a decrease in postoperative mortality over the 15-year period.

Regarding the type of operation, we were aware of the potential confounding by indication. We did address this in the discussion when interpreting our results. We acknowledge that the type of surgery is chosen based on type of fracture, patient age, comorbidities, and daily level of function in order to give the patient the best outcome (Claus et al. 2008). In order to reduce confounding by indication, we adjusted for age and comorbidities in our multivariate analysis. Despite this adjustment, we still observed a statistically significant association between type of surgery and mortality.

In this study, we chose to display the primary and first surgical intervention for hip fracture patients. However, we recognize that for patients with reposition as the first surgical intervention, the secondary surgical intervention could have been included in our analysis. Regarding choice of reference group in Table 2, we do not find it essential to change this for type of operation since hazard ratios and 95% confidential intervals are stated for each type of operation.

We acknowledge the extensive research on hip fracture surgery that has been done in the past 25 years, as pointed out by Dr Rogmark. With our paper, we bring epidemiological data on outcome after hip fractures over a 15-year period from Denmark into the broad discussion regarding enhanced recovery after surgery. Our goal is to encourage a debate on how to improve recovery and mortality after surgery.

**Ossian Gundel**

Center for Surgical Science, Department of Surgery,

Zealand University Hospital, Koege, Denmark

*E-mail:* qrb124@alumni.ku.dk
